# M. Jobert De Lamballe

**Published:** 1845-09

**Authors:** 


M. Jobert de Lamballe, surgeon to the Hôpital Saint Louis, Paris, has been appointed surgeon to the Hôtel Dieu, to fill up the vacancy caused by the death of M. Breschet.—*Prov. Med. and Surg. Journ.*

